# Normal sleep on mechanical ventilation in adult patients with congenital central alveolar hypoventilation (Ondine’s curse syndrome)

**DOI:** 10.1186/s13023-017-0569-5

**Published:** 2017-01-23

**Authors:** Valérie Attali, Christian Straus, Michel Pottier, Marie-Annick Buzare, Capucine Morélot-Panzini, Isabelle Arnulf, Thomas Similowski

**Affiliations:** 1 Sorbonne Universités, UPMC Université Paris 06, INSERM, UMRS1158 “Neurophysiologie Respiratoire Expérimentale et Clinique”, Paris, France; 2 Service des Pathologies du Sommeil, Hôpital Pitié-Salpêtrière, 47-83 Boulevard de l’Hôpital, 75651 Paris, Cedex 13 France; 3 Branche “Adultes” du Centre de Référence du Syndrome d’Ondine, F-75013 Paris, France; 4 Service d’Explorations Fonctionnelles Respiratoires de l’Exercice et de la Dyspnée EFRED, F-75013 Paris, France; 5 Unité ambulatoire d’Assistance Respiratoire à Domicile, F-75013 Paris, France; 6 Service de Pneumologie et Réanimation Médicale, Département R3S, Hopitaux Universitaires Pitié-Salpêtrière Charles Foix, AP-HP, F-75013 Paris, France; 7 Brain Research Institute-UPMC Paris 6 Univ Inserm U 1127, CNRS UMR 7225, Paris, France

**Keywords:** Congenital central hypoventilation syndrome, Ondine’s curse syndrome, Sleep structure, Polysomnography, Slow wave sleep, Local sleep, Mechanical ventilation

## Abstract

**Background:**

The purpose of this study was to describe the sleep structure (especially slow wave sleep) in adults with congenital central hypoventilation syndrome (CCHS), a rare genetic disease due to mutations in the PHOX2B gene. Fourteen patients aged 23 (19.0; 24.8) years old (median [1^rst^-3rd quartiles]) with CCHS underwent a sleep interview and night-time attended polysomnography with their ventilatory support. Their sleep variables were compared to those collected in 15 healthy control subjects matched for age, sex and body mass index.

**Results:**

The latency to N3 sleep was shorter in patients (26.3 min [24.0; 30.1]) than in controls (49.5 min [34.3; 66.9]; *P* = 0.005), and sleep onset latency tended to be shorter in patients (14.0 min [7.0; 20.5]) than in controls (33.0 min [18.0; 49.0]; *P* = 0.052). Total sleep time, sleep stage percentages, sleep fragmentation as well as respiratory and movement index were within normal ranges and not different between groups.

**Conclusions:**

Normal sleep in adult patients with CCHS and adequate ventilator support indicates that the PHOX2 gene mutations do not affect brain sleep networks. Consequently, any complaint of disrupted sleep should prompt clinicians to look for the usual causes of sleep disorders, primarily inadequate mechanical ventilation. Shorter N3 latency may indicate a higher need for slow wave sleep, to compensate for the abnormal respiratory-related cortical activity during awake quiet breathing observed in patients with CCH.

## Background

Congenital central hypoventilation syndrome (CCHS) is a rare genetic disease caused by mutations of the PHOX2B gene [1]. The syndrome is associated with multiple autonomic nervous system disorders. Patients with CCHS have profoundly impaired sensitivity to carbon dioxide and suffer from profound sleep-related hypoventilation. Consequently, right from birth, their survival depends on mandatory ventilatory assistance during sleep. Clinical practice guidelines stipulate that polysomnography should be performed in patients with CCHS on a regular basis, twice-yearly then yearly, to assess ventilatory needs in all sleep stages [2]. However, no published data are available concerning sleep structure and sleep quality in patients with CCHS, although several factors could impact sleep architecture, including mechanical ventilation-related sleep alterations (e.g., giving rise to frequent alarms), as well as putative neurodevelopmental abnormalities due to the genetic defect, as observed in other genetic disorders, [3] changes in circadian rhythms (as already documented in children with CCHS [4]), or chronic anxiety (frequent in adult patients with CCHS [5]). In addition, adult patients with CCHS exhibit respiratory-related motor cortical activity during awake resting breathing, which is absent in normal individuals [6]. This activity is interpreted as a cooperative wake-related cortical mechanism supplementing the deficient medullary ventilatory drive [6]. This hypothesis has recently been supported by the observation of similar cortical activity in normal subjects continuing to breathe despite experimental hypocapnia, namely in a situation in which medullary ventilatory drive is reduced [7]. Because activation of neuronal populations during wakefulness may be followed by higher local sleep in the corresponding cortical areas (as shown by increased local slow wave sleep activity after a localized learning task [8] or after excitatory repetitive transcranial magnetic stimulation [9]), the respiratory-related cortical activity observed in CCHS could increase slow wave sleep. Consequently, this study was designed to compare sleep structure (especially slow wave sleep) in adult patients with CCHS vs. healthy controls.

## Methods

### Participants

Fourteen of the 17 adult patients with CCHS followed at the adult branch of the French national reference center for this disease at the time of the study were included (age: 23 [22; 26] years old, median [1rst-3rd quartile]). To ensure a homogeneous study population comparable to the control population, three patients from the adult CCHS cohort were not included (two patients with late onset CCHS -one woman aged 49 and one man aged 37 at the time of the diagnosis-, and one 27-year-old patient with a neonatal diagnosis of CCHS -frameshift mutation- but documented anoxic brain lesions and severe psychiatric disorders requiring psychotropic treatment most likely to affect sleep). All included patients had been diagnosed with CCHS soon after birth and, for the first 18 years of life, had been followed-up at least annually by the French reference center for CCHS, in addition to their usual care and home mechanical ventilation management. PHOX2B abnormalities consisted of alanine expansion in 13 cases (25 alanine, *n* = 5; 26, *n* = 3; 27, *n* = 2; 29, *n* = 1; 30, *n* = 1 and 31, *n* = 1) and frameshift mutation in 1 case. None of the patients had a history of cor pulmonale or polycythemia or any other evidence suggesting insufficient ventilatory support. Serum hemoglobin was 13.1 [12.6–13.6] g/100 ml at the time of polysomnography. None of the patients included in the study had gross evidence of neurocognitive delay. All patients had completed a normal schooling program in the French system since early childhood. At the time of the study, six were still attending secondary school, five had graduated at the end of high school, and 3 were university students. None of the participating patients took medications likely to impair sleep structure. Four patients were still tracheotomized and ten had been transitioned to noninvasive ventilation. One patient suffered from permanent hypoventilation requiring 24-h-a-day ventilatory assistance (diaphragm pacing during the day, mechanical ventilation during the night). All other patients were ventilated only during sleep, but three of them had documented diurnal hypercapnia. All patients had undergone at least one previous in-lab polysomnography, whether as children or as adults. They were informed that their clinical parameters collected during routine clinical care could be used for research purposes and they did not refuse this procedure. Fifteen healthy subjects matched for age, gender and body mass index and devoid of any sleep complaints were recruited by word of mouth during the same period, signed an informed consent and received financial compensation. They were selected for Epworth sleepiness scale ≤ 10, absence of any sleep complaints, absence of chronic sleep deprivation, and absence of chronic use of sedative drugs or alcohol. They were free of any respiratory disease. The protocol for patients was approved by the ethics committee of the National Sleep Medicine Society (*Société Française de Recherche et Médecine du Sommeil)*. Data concerning healthy subjects were extracted from another sleep study, in which they were entered as controls and which had been approved by an ethics committee (Comité de protection des personnes - Ile de France VI, Paris, France) [10].

### Sleep monitoring

Patients and controls were interviewed about sleep symptoms and completed the Epworth sleepiness scale [11]. Sleep monitoring included EEG (Fp1/A2, C3/A2, C3/01), left and right electro-oculograms, surface electromyogram of mentalis and left and right tibialis anterior muscles, respiratory flow at the mask for patients and nasal pressure plus tracheal sounds for controls, ECG, transcutaneous oxygen saturation, thoracic and abdominal efforts through piezoelectric belts and position monitoring. Patients underwent a single night-time attended polysomnography with their ventilatory support. The adequacy of the ventilatory support settings was documented during the polysomnography by the absence or quasi-absence of alarms, the absence of episodes of mask disconnection in patients receiving noninvasive mechanical ventilation, the absence of significant respiratory events (leaks or obstruction), and the absence of desaturation episodes. The mean SpO_2_ during sleep was 97.5% [96.6; 98.1] and the time with SpO_2_ below 90% was 0.0% [0.0; 0.2].

The corresponding control subjects were studied on two consecutive nights. During the first night, respiratory variables were recorded together with EEG, which demonstrated the absence of sleep-disordered breathing. Their apnea-hypopnea index was 0.9 [0.0; 1.8], mean SpO_2_ during sleep was 96.4% [96.1; 97.6] and the time with SpO_2_ below 90% was 0.0% [0.0; 0.0]. During the second night, designed to compare controls with CCHS patients, in order to attenuate the influence of the first night effect, the respiratory sensors were removed to improve comfort. Given the high repeatability of the night-to-night apnea-hypopnea index in normal subjects [12], it is reasonable to assume that respiratory events were not more frequent during the second night than during the first night.

Sleep stages, and motor and respiratory events were scored by a single experienced scorer blinded to the status (patient or control) of the participants (MAB), according to international criteria [13].

### Statistical analysis

Data were expressed as median and interquartile range. Between-group comparisons were performed using the Mann-Whitney test for continuous measures (anthropometric and sleep data) and Fisher’s exact test for gender, using online university software resources (biostatgv.sentiweb.fr). A P value less than 0.05 was considered statistically significant.

## Results

No significant difference was observed between patients and controls in terms of gender, age, body mass index, and score on the Epworth sleepiness scale (Table 1). No patient or healthy subject complained of insomnia, restless legs syndrome, nightmares, nocturia, morning headache, morning fatigue, daytime fatigue, excessive daytime sleepiness and need for a daytime nap. Sleep structure was normal in both groups. The latency to sleep onset tended to be shorter in patients than in controls, (*P* = 0.052). The latency to N3 sleep was shorter in patients than in controls (*P* = 0.005). No other difference was observed in terms of sleep duration, continuity, architecture, and fragmentation, as well as periodic leg movement index. The three patients exhibiting diurnal hypercapnia had normal hypnograms. The N1 percentages were 2.8, 5.2 and 4% of total sleep time, respectively. The N2 percentages were 48.8, 49.7 and 45.6%. The N3 percentages were 34.3, 25.7 and 27.5%. The REM percentages were 14.2, 19.5 and 22.9%. Mean SaO_2_ during sleep (97.7, 98.1, and 98.5%, respectively of), and minimal SaO_2_ (91, 95 and 98%, respectively) were in the adequate range.

**Table 1:** Demographic, clinical, respiratory and sleep variables in patients with congenital central hypoventilation syndrome (CCHS) and healthy controls

	Patients with CCHS	Healthy Controls	*P* value
Number	14	15	
Gender, F/M	9/5	11/4	0.70
Age, y	23.0 (19.0; 24.8)	23.0 (22.0; 26.0)	0.42
Body mass index, kg/m^2^	21.7 (20.7; 24.0)	21.7 (20.1; 22.8)	0.95
Epworth sleepiness score, 0-24	7 (2; 8)	6 (4; 7)	0.73
NIV/Tracheotomy	10/4	NA	NA
Blood gases			
pH	7.42 (7.38; 7.44)	NA	NA
PaCO_2_, mmHg	40 (34; 44)	NA	NA
PaO_2_, mmHg	101 (86: 112)	NA	NA
Awake SpO_2_, %	98 (96; 99)	NA	NA
Total sleep period, min	514.5 (489.5; 585.8)	552.0 (522.5; 588.5)	0.47
Total sleep time, min	484.0 (432.8; 512.3)	500.0 (446.5; 540.0)	0.42
Sleep efficiency, %	93.7 (82.9; 95.1)	92.9 (88.6; 95.0)	0.86
Latency to, min			
Sleep onset	14.0 (7.0; 20.5)	33.0 (18.0; 49.0)	0.052
N3	26.3 (24.0; 30.1)	49.5 (34.3; 66.9)	0.005
REM sleep	64.5 (52.3; 81.5)	64.0 (56.5; 69.0)	0.95
Sleep stages, min			
N1	17.5 (10.5; 25.5)	26.0 (15.5; 32.0)	0.21
N2	209.5 (187.3; 247.0)	223.0 (190.0; 245.5)	0.65
N3	117.0 (109.8; 133.8)	120.0 (106.0; 142.0)	0.83
REM sleep	110.0 (90.3; 122.3)	126.0 (109.0; 133.0)	0.11
Arousals (/h)	13.3 (8.5; 17.4)	11.5 (9.2; 15.2)	0.79
Periodic leg movements (/h)	0.1 (0.0; 3.3)	0.2 (0.0; 0.7)	0.95

*NA* not available, *NIV* noninvasive ventilation, Values are median (interquartile range)

## Discussion

This study (seemingly the first to describe the sleep structure in adult patients with CCHS) shows that these patients, when adequately ventilated, do not exhibit any sleep abnormalities according to a standard polysomnography approach. Normal sleep in this genetically homogeneous CCHS cohort suggests that the genetic PHOXB2 defect does not directly impact the brain structures responsible for sleep and wake. Autonomic dysregulation during sleep was not assessed in this study, and we therefore cannot exclude the hypothesis that autonomic abnormalities such as hypothermia occurred in the CCHS group. However, such abnormalities apparently did not affect sleep architecture. Failure to achieve adequate mechanical ventilation during sleep in chronic respiratory or neurorespiratory diseases requiring ventilatory support during sleep is associated with impaired sleep efficiency and architecture [14, 15]: by analogy, CCHS patients reporting sleep complaints should be primarily investigated for adequate mechanical ventilation. Of note, the majority of patients (ten patients) were using noninvasive ventilation (the four remaining patients were still tracheotomized), which is an effective modality to treat acute respiratory distress in children with central nervous system disorders [16], and is recommended after the age of 6 to 8 years and in adults in patients with stable CCHS [2], and all patients had optimal respiratory support.

Although situated in the normal range in both groups, the latency to N3 sleep was shorter in patients than in controls, and sleep onset latency tended to be shorter in patients. A reduction of sleep onset and N3 latencies has been related to improved sleep continuity and quality in other studies [17]. These signs of excellent sleep in CCHS suggest that patients relaxed more rapidly (shorter sleep onset latency) than controls and that their cortical neurons slowed and synchronized more rapidly during sleep (shorter N3 sleep latency). A “first night effect” (which affects sleep onset latency, as well as REM sleep onset latency and sleep efficiency) could artificially prolong the sleep onset latency in controls (who are less used to sleeping with equipment), but not in patients (who are used to sleeping with mechanical ventilation since birth and who regularly undergo sleep and oxygen saturation monitoring). However, this effect was unlikely, as the comparison was based on the second night in controls, and in view of the absence of any other evidence of impaired sleep (normal and similar sleep efficiency, duration, structure, as well as normal REM sleep latency and arousal index). Alternatively, patients with CCHS could be more isolated from noises and external disturbances that prolong sleep onset latency. In this respect, children with CCHS have a dysfunction of brainstem auditory pathways [18], which may contribute to “sensory isolation” during sleep and reduce the ability of the brain to monitor signals despite their possibly vital importance (e.g., ability to detect ventilator alarms). However, this speculative interpretation is not supported by a lower arousal index in patients with CCHS.

The more rapid installation of N3 sleep (and rapid synchronization and slowing down of cortical neuron activity) may indicate a greater need for sleep in patients with CCHS. The need to use a cortical network to breathe during wakefulness could possibly be compensated by enhanced restoration of the same cortical network during sleep under mechanical ventilation, as patients with CCHS exhibit abnormal respiratory-related cortical activity during awake quiet breathing [6], which could result in a “compensatory” increase in slow wave sleep [8, 9]. However, N3 time was normal and similar in the CCHS and control groups. It would be interesting to determine whether more subtle signs of cortical restoration, such as diffuse or localized changes in the EEG power spectrum (e.g., increases in delta power), are observed in these patients. However, the routine EEG montage was too limited to allow this spatial analysis. The small size of our sample also prevented us from drawing any other conclusions. This limitation is inherent to any exceptional disease, but is partially compensated in this case by the genetic and age homogeneity of the CCHS group.

## Conclusion

From a pragmatic point of view, this study shows that sleep is basically normal in adult patients with CCHS when they are adequately ventilated. This is reassuring information for patients and their families. It also means that any sleep complaint should prompt clinicians to look for abnormalities unrelated to the genetic disorder itself, primarily inadequate control of alveolar ventilation, regardless of its mechanisms.

## Abbreviations

CCHS: Congenital central hypoventilation syndrome
